# PEDF Regulates Vascular Permeability by a γ-Secretase-Mediated Pathway

**DOI:** 10.1371/journal.pone.0021164

**Published:** 2011-06-17

**Authors:** Jun Cai, Lin Wu, Xiaoping Qi, Sergio Li Calzi, Sergio Caballero, Lynn Shaw, Qing Ruan, Maria B. Grant, Michael E. Boulton

**Affiliations:** 1 Department of Anatomy and Cell Biology, University of Florida, Gainesville, Florida, United States of America; 2 Department of Pharmacology and Therapeutics, University of Florida, Gainesville, Florida, United States of America; Istituto Dermopatico dell'Immacolata, Italy

## Abstract

Increased vascular permeability is an inciting event in many vascular complications including diabetic retinopathy. We have previously reported that pigment epithelium-derived factor (PEDF) is able to inhibit vascular endothelial growth factor (VEGF)-induced angiogenesis through a novel γ-secretase-dependent pathway. In this study, we asked whether inhibition of VEGF-induced permeability by PEDF is also γ-secretase-mediated and to dissect the potential mechanisms involved. Vascular permeability was assessed *in vitro* by measuring transendothelial resistance and paracellular permeability to dextran and *in vivo* by following leakage of intravenous FITC-labelled albumin into the retina in the presence or absence of VEGF and PEDF in varying combinations. Experiments were undertaken in the presence or absence of a γ-secretase inhibitor. To assess junctional integrity immunohistochemistry for the adherens junction (AJ) proteins, VE-cadherin and β-catenin, and the tight junction (TJ) protein, claudin-5 was undertaken using cultured cells and flat mount retinas. Protein expression and the association between AJ proteins, VEGF receptors (VEGFRs) and γ-secretase constituents were determined by immunoprecipitation and Western Blot analysis. In selected experiments the effect of hypoxia on junctional integrity was also assessed. PEDF inhibition of VEGF-induced permeability, both in cultured microvascular endothelial cell monolayers and *in vivo* in the mouse retinal vasculature, is mediated by γ-secretase. PEDF acted by a) preventing dissociation of AJ and TJ proteins and b) regulating both the association of VEGF receptors with AJ proteins and the subsequent phosphorylation of the AJ proteins, VE-cadherin and β-catenin. Association of γ-secretase with AJ proteins appears to be critical in the regulation of vascular permeability. Although hypoxia increased VEGFR expression there was a significant dissociation of VEGFR from AJ proteins. In conclusion, PEDF regulates VEGF-induced vascular permeability via a novel γ-secretase dependent pathway and targeting downstream effectors of PEDF action may represent a promising therapeutic strategy for preventing or ameliorating increased vascular permeability.

## Introduction

The vascular endothelium regulates fluid and solute exchange between the blood and interstitial space [1], [2]. Increased vascular permeability and vascular leakage by paracellular routes are usually pathological and involved in conditions as diverse as cancer, ischemic heart disease, asthma, subcutaneous edema, inflammation, wound healing and diabetic microvascular complications [3], [4], [5]. Furthermore, diabetic macular edema, which involves the breakdown of the blood–retinal barrier, also occurs and is responsible for a major part of vision loss, particularly in Type 2 diabetes [6]. Paracellular permeability is mediated by the co-ordinated opening and closing of endothelial cell-cell junctions [7], [8]. These transmembrane complexes occur as tight junctions (TJs) and adherens junctions (AJs) of which the latter, unlike epithelial cells, predominate in endothelial cells [2], [8]. The blood barrier requires the adhesive properties of VE-cadherin and claudin-5 which are key components of the AJs and TJs respectively. The loss of AJ and TJ integrity between endothelial cells leads to increased solute flux [9], [10]. Moreover, unlike epithelial cells, AJs and TJs are intermingled in microvascular endothelial cells to form a complex zonular system and adherens junctions are reported to regulate the organization and stability of tight junctions [7], [8].

Vascular endothelial growth factor (VEGF), originally termed vascular permeability factor, is a key factor in the pathogenesis of vascular complications including increased retinal vascular permeability which is a hall mark of early diabetic retinopathy [4], [11]. In animal studies, intravitreal injection of VEGF results in a rapid and sustained increase in vascular permeability in the retina [12], [13], [14]. Furthermore, a variety of VEGF-blocking strategies have confirmed the critical role of VEGF in the permeability response of diabetic animal models [14], [15], [16], [17] and have shown a reduction in vascular permeability in patients with diabetic macular edema [18], [19], [20], [21]. Studies of the mechanisms by which VEGF induces increases in vascular permeability indicate that multiple pathways are involved [4]. The VEGF family consists of many members of which VEGFA is considered the most dominant in angiogenesis. VEGFA interacts with VEGF receptor (VEGFR) 1 and 2 on vascular endothelial cells [22], [23]. VEGFR2 signals via a strong tyrosine kinase signal which is able to regulate endothelial function and survival via a number of different signalling pathways [23]. By contrast, VEGFR1 has a higher affinity for VEGF than VEGFR2 but only has weak kinase activity. A growing body of evidence supports that VEGFR1 is a potent regulator of VEGFR2 action [24], [25]. Interestingly, VEGFR2 is closely associated with VE-cadherin in AJs and has been implicated in phosphorylation of proteins in the AJ complex [26], [27]. However, the role of VEGFR1 in the regulation of endothelial junction integrity has received little attention.

Pigment epithelium-derived factor (PEDF) is a 50-kDa non-inhibitory member of the serine protease inhibitor (Serpin) gene family, and a potent antiangiogenic factor [28], [29]. PEDF is secreted by many retinal cells including the Müller cells, vascular endothelial cells, pericytes and retinal pigment epithelial cells and has been reported to inhibit neovascularization in animal models for diabetes, prevent the accumulation of advanced glycation endproducts, reduce oxidative damage and reduce inflammation [30], [31], [32], [33], [34]. Furthermore, vitreous levels of PEDF are significantly reduced in diabetic macular edema [35], [36]. Numerous studies have demonstrated that PEDF inhibits VEGF- and stress-induced vascular permeability both in vitro and in vivo [34], [37], [38], [39], [40], [41], [42] but the mechanisms are poorly understood and no definitive receptor has been identified for PEDF. To date, only two binding partners have been identified for PEDF: a lipase-linked membrane protein and a laminin receptor [29], [43]. We have previously reported that PEDF a) is down regulated in the diabetic retina and tumors [44], [45], b) inhibits growth factor-induced angiogenesis in microvascular endothelial cells, c) regulates the intracellular translocation of both intact and cleaved VEGFR1 and c) blocks VEGF-induced phosphorylation of VEGFR-1 [24], [46], [47]. These events were dependent on γ-secretase which is a complex composed of four different integral proteins (presenilin, nicastrin, Aph-1 and Pen-2) [46]. The most studied components of the γ-secretase complex are presenilin, which is an integral enzyme in the transmembrane cleavage of substrates and nicastrin that is purported to be essential for substrate recognition [46].

In this study we show that PEDF blocks VEGF-induced vascular permeability by preventing dissociation of endothelial AJs and TJs via a γ-secretase-dependent mechanism. We further demonstrate that PEDF regulates both the association of VEGF receptors with AJ proteins and phosphorylation of the AJ proteins, VE-cadherin and β-catenin. The association of γ-secretase with AJ proteins appears to be critical in the regulation of vascular permeability. Finally, we show that hypoxia, which is a feature of pathological angiogenesis, both upregulates VEGFR expression and increases dissociation of VEGFRs from AJ proteins. This represents a novel γ-secretase-dependent pathway that regulates vascular permeability.

## Results

### PEDF blocks VEGF-induced vascular permeability by preventing dissociation of endothelial AJs and TJs via a γ-secretase-dependent mechanism

**Figure 1:**
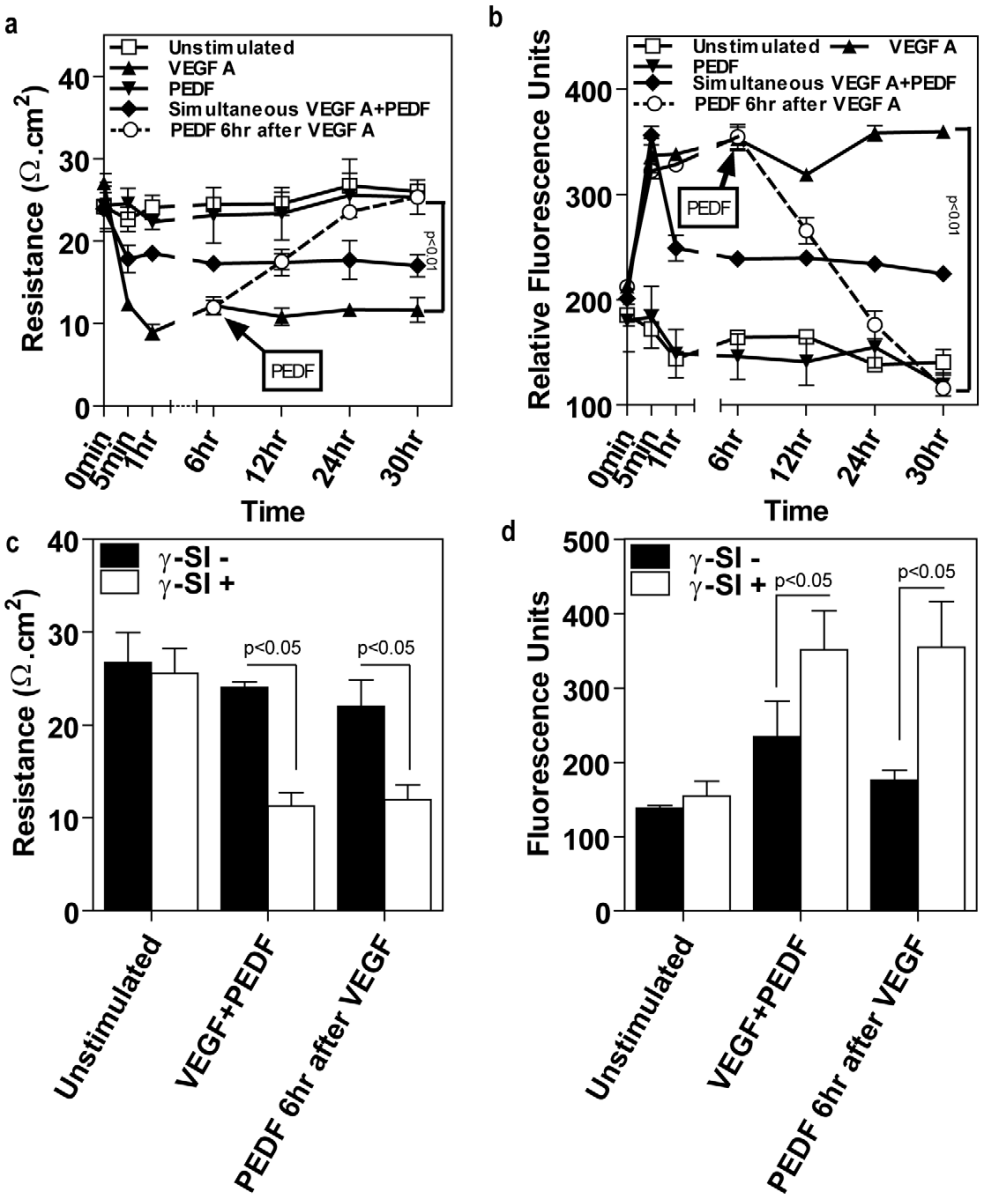
PEDF blocks microvascular endothelial permeability via a γ-secretase dependent mechanism. (**a**) Temporal changes in transendothelial resistance across a microvascular endothelial monolayer grown on a Transwell insert treated with vehicle (unstimulated), VEGFA alone, PEDF alone, simultaneous VEGFA+PEDF, PEDF 6 hours post VEGF (n = 4 independent experiments). VEGFA and PEDF were used at 100 ng/ml. (**b**) Paracellular macromolecular permeability to 40 kDa Dextran-FITC using the conditions described in (A) (n = 4). In the case of PEDF 6 hours post VEGF, Transwell inserts were transferred to new wells containing basal medium without fluorescent dextran. (**c**) and (**d**) The effect of γ-secretase inhibitor (γ-SI) on PEDF reduction of VEGF-induced endothelial permeability; (**c**) TER, (**d**) paracellular flux. γ-secretase inhibitors were used at 1 nM and data is shown for L685485. Data are represented as means (n = 4) ± SEM.

Addition of VEGF-A to cultured retinal microvascular endothelial cells caused a significant 62% decrease in TER and 130% increase in the transendothelial flux of flourescent dextran which was sustained over a 24 hour period compared to untreated control, cells treated with PBS alone, PEDF alone or γ-secretase inhibitor alone (Figs. 1a, b). However, PEDF significantly reduced VEGF-induced permeability and this was dependent on time of application. Cells treated with PEDF in combination with VEGF at time 0 showed a significant ∼50% decrease in VEGF-induced permeability (Figs. 1a, b). However, this was not as great as that observed when PEDF was applied 6 hours following VEGF. Addition of PEDF post VEGF treatment resulted in complete reversal of VEGF-induced permeability by 24 hours compared to cells treated with VEGF alone. Higher concentrations of PEDF did not further decrease VEGF-induced permeability (data not shown) [24]. In all cases, γ-secretase (which we have previously shown inhibits PEDF signaling [24]) blocked the inhibitory effect of PEDF on VEGF-induced permeability (Fig. 1c, 1d).

**Figure 2:**
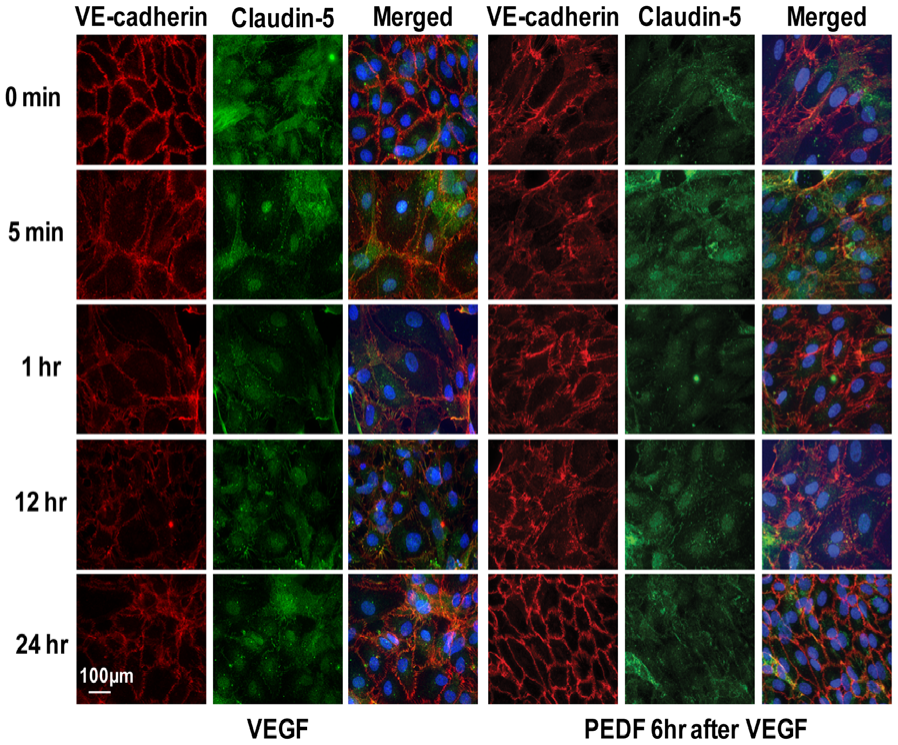
The temporal response of AJs and TJs to VEGF or VEGF plus PEDF. Representative pictures of confluent cultures of microvascular endothelial cells treated with VEGFA alone or VEGFA followed by PEDF 6 hours later triple stained for VE-cadherin (red), claudin-5 (green) and nuclei (DAPI) and assessed at different times over 24 hours using confocal microscopy. VEGFA and PEDF were used at 100 ng/ml. Scale bar = 100 µm.

**Figure 3:**
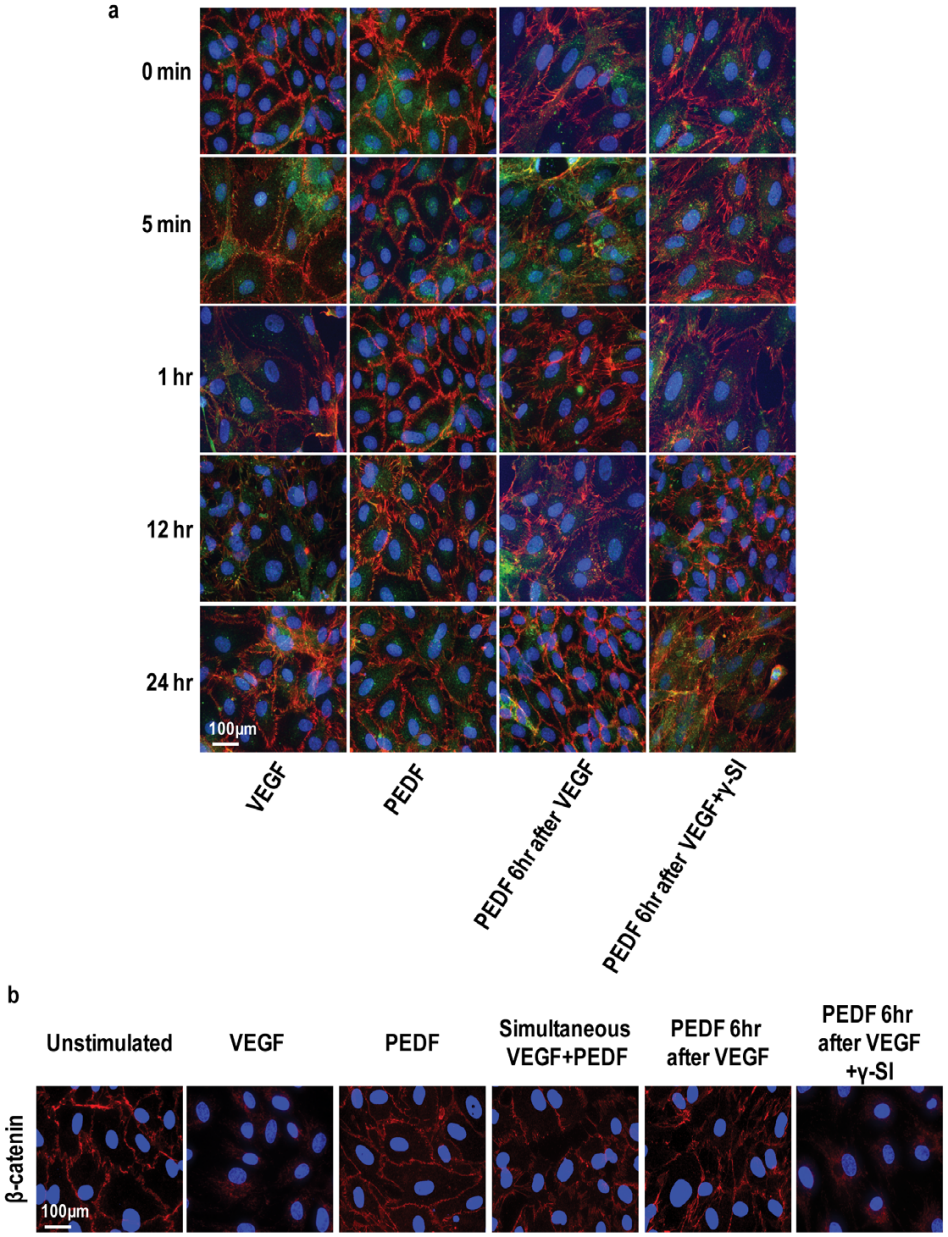
PEDF prevents VEGF-induced dissociation of endothelial AJs and TJs. Representative immunofluorescent images of confluent cultures of microvascular endothelial cells treated with vehicle (unstimulated), VEGFA alone, PEDF alone, simultaneous VEGFA+PEDF, PEDF 6 hours post VEGF and PEDF 6 hours post VEGF+γ-secretase inhibitor (γ-SI) (n = 4 independent experiments). VEGFA and PEDF were used at 100 ng/ml and γ-secretase inhibitor at 1 nM. (**a**) Cultures were triple stained for VE-cadherin (red), claudin-5 (green) and nuclei (DAPI, blue) and assessed at different times over 24 hours using confocal microscopy. Merged images are shown with colocalization of VE-cadherin and claudin-5 appearing as yellow. (**b**) The effect of PEDF on β-catenin using the conditions described in (A) (n = 4). Scale bar = 100 µm.

Immunohistochemistry demonstrated lateral membrane localization of VE-cadherin and claudin-5 in endothelial cell monolayers and this was unaffected by addition of PEDF alone (Fig. 2) or γ-secretase inhibitor alone. The vehicle control remained identical to VE-cadherin and Claudin-5 staining at time 0 throughout the time course of the experiment. However, VEGF alone led to a rapid disassociation of the cell-cell contacts and a major loss of staining for VE-cadherin at the junctional complexes within 15 minutes and reduced staining for claudin 5 observed at 1 and 12 hours post treatment (Fig. 2). Addition of PEDF in combination with VEGF (data not shown), or PEDF 6 hours post VEGF treatment, prevented the VEGF-induced dissociation of lateral junctional complexes (Fig. 3). However, γ-secretase inhibition, blocked the inhibitory effect of PEDF on VEGF-induced junctional changes. β-catenin, which is associated with the integrity of VE-cadherin in adherens junction [8], was dissociated by VEGF and translocated to both the cytosolic and nuclear compartments and this VEGF-induced dissociation was prevented by PEDF (Fig. 3). Inhibition of γ-secretase prevented the PEDF-induced effect on VEGF dissociation of β-catenin.

**Figure 4:**
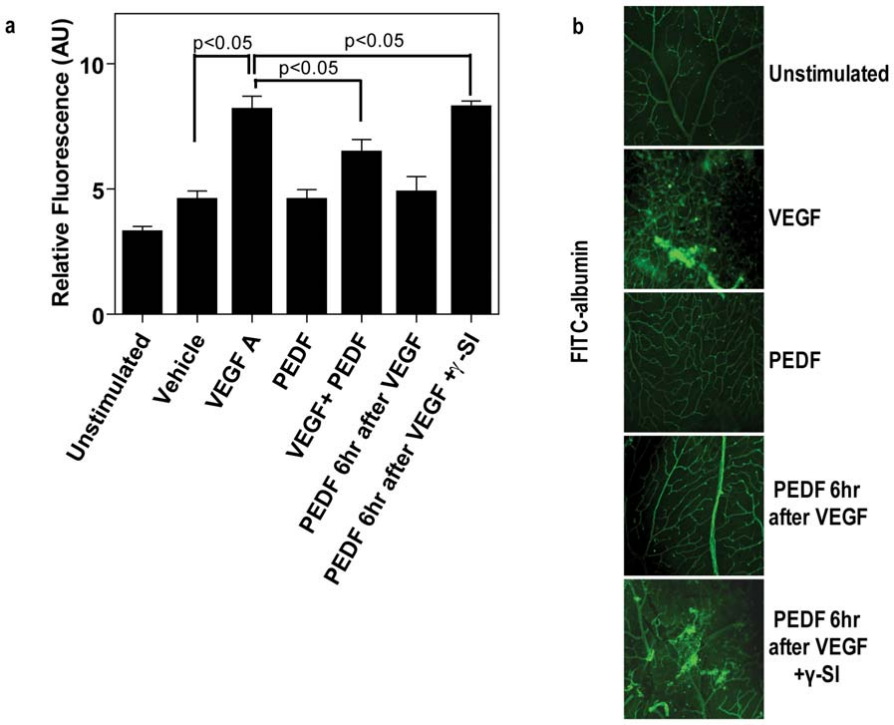
PEDF blocks VEGF-induced retinal vascular permeability in mice. C57BL/6 mice received intravitreal injections of vehicle (unstimulated), VEGFA alone, PEDF alone, simultaneous VEGFA+PEDF, PEDF 6 hours post VEGF and PEDF 6 hours post VEGF+γ-secretase inhibitor (γSI) (n = 18 animals per treatment). Test compounds (1 µl per eye were given at the following concentrations, VEGF 80 ng/µl; PEDF (80 ng/µl); L685485 24 ng/µl; DAPT 16 ng/µl. 46 hours post the first injection mice received tail vein injections of FITC-labeled albumin. Uninjected animals acted as the baseline control. (**a**) 46 hours post the first injection mice received tail vein injections of FITC-labeled albumin and retinas were taken for analysis 2 hours later (n = 10–20 per group). Leakage of systemic FITC-labeled albumin into the retina was assessed by measuring total fluorescence in homogenized retina using a fluorescence plate reader. Control is the baseline fluorescence in untreated animals. Data are represented as means ± SEM. (**b**) Representative confocal microscopy showing dilated vessels and leakage of FITC-labeled albumin in the retinas of mice treated with vehicle (unstimulated), VEGF, PEDF, PEDF 6 hours post VEGF and PEDF 6 hours post VEGF+γ-secretase inhibitor (γ-SI). Scale bar = 50 µM.

To confirm that the ability of PEDF to inhibit VEGF-induced permeability was not limited to cell culture, we repeated these studies in mice. Intravitreal injection of VEGF in C57BL/6 mice resulted in significant intraretinal leakage of systemically introduced fluorescent albumin (Fig. 4). However, intravitreal injection of PEDF either together with VEGF or 6 hr following VEGF resulted in significant inhibition of VEGF-induced fluorescent albumin leakage into the retina and this was greatest when PEDF was injected 6 hours following VEGF (Fig. 4a). γ-Secretase inhibition blocked the inhibitory effect of PEDF on VEGF-induced permeability (Fig. 4a). Confocal microscopy of flat mount retinal preparations showed intraretinal fluorescent albumin in greater than 90% of the retina in VEGF treated animals, confirming increased vascular leakage compared to vehicle only controls, PEDF alone or γ-secretase inhibitor alone (Fig. 4b). By marked contrast, minimal vascular leakage of fluorescent albumin was observed in animals receiving PEDF and VEGF and in all cases, γ-secretase inhibition blocked the inhibitory effect of PEDF on VEGF-induced permeability (Fig. 4b).

**Figure 5:**
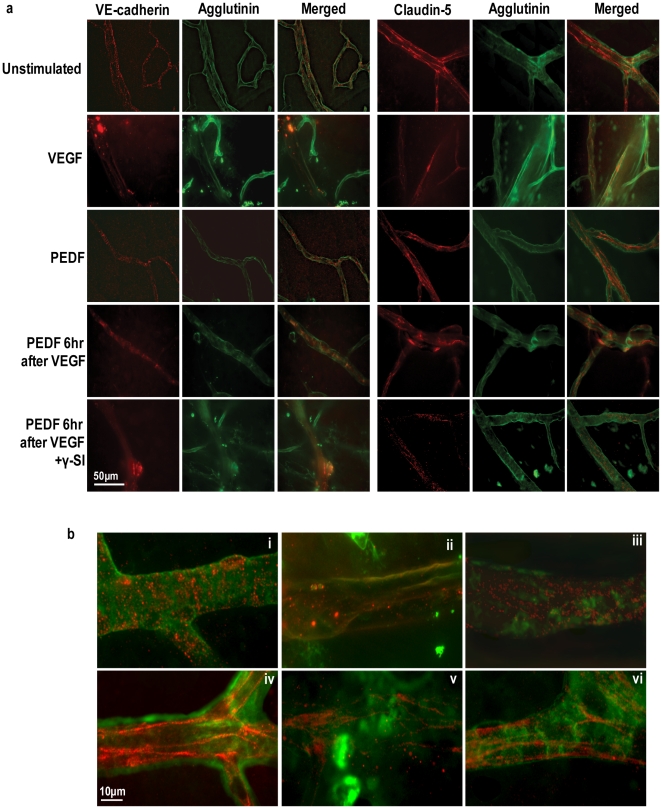
PEDF prevents VEGF-induced dissociation of endothelial AJs and TJs in the retinal vasculature of mice. (**a**) Representative confocal images of retinal vessels in flat mount preparations from unstimulated and animals, treated as described in Figure 4, immunostained with VE-cadherin or claudin-5 (red) and FITC-conjugated agglutinin (green) to visualize retinal vessels. Merged images are shown with colocalization of VE-cadherin and claudin-5 appearing as yellow. Scale bar = 50 µM. The lower panel (**b**) shows representative merged higher power images of retinal vessels stained for VE-cadherin (i–iii) or claudin-5 (iv–vi) (red) and FITC-conjugated agglutinin (green). *i, iv = unstimulated; ii, v = VEGF treatment; iii, vi = PEDF 6 hr after VEGF.* Scale bar = 10 µM.

Using immunohistochemistry we stained for either VE-cadherin or claudin 5 to assess the spatial relationship between AJ and TJ protein expression and changes in paracellular vascular permeability (Fig. 5). A typical staining for an intact vasculature will show clearly demarcated lateral membranes of microvascular endothelial cells. This was observed for both VE-cadherin and claudin-5 in mouse eyes without injection or with intravitreal injection of the PBS vehicle (Fig. 5a). Localization and integrity of junctional complexes were not changed by exposure to PEDF alone or γ-secretase inhibitor alone over a 48 hour period. However, intravitreal injection of VEGF resulted in an almost complete loss of staining of the junctional network for greater than 90% of the retinal vessels indicative of loss of junctional complexes and this was confirmed by excessive leakage of fluorescent albumin into the retina (Fig. 5). By contrast, eyes which had received PEDF simultaneously or 6 hrs after VEGF-induced vascular permeability exhibited a pattern of VE-cadherin and claudin 5 staining similar to that seen for controls in greater than 75% of the retina although a few areas remained in which junctional complexes seemed to be less well formed. The beneficial effect of PEDF on VEGF-induced permeability was blocked by inhibition of γ-secretase (Fig. 5).

### PEDF regulates the association of VEGF receptors with AJ proteins

**Figure 6:**
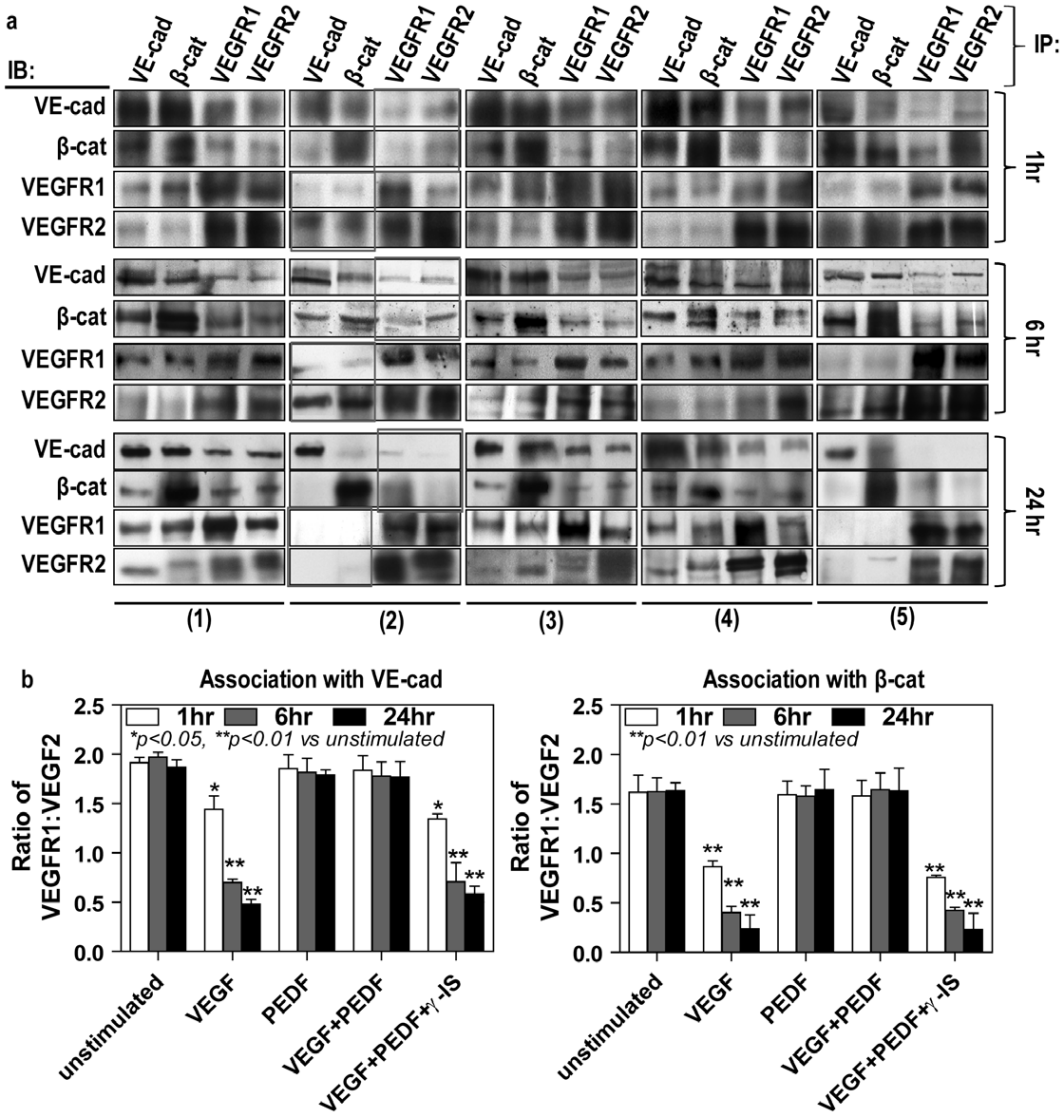
PEDF maintains the association between AJ proteins and VEGF receptors in VEGF treated cells. Confluent retinal endothelial cells were either unstimulated (1) treated with VEGF alone (2), PEDF alone (3), VEGF followed by PEDF 6 hours later without (4) and with (5) γ-secretase inhibitor for 24 hours. VEGFA and PEDF were used at 100 ng/ml and γ-secretase inhibitor at 1 nM. Total cell lysates were split into four equal portions and immunoprecipitated with anti-VE-cadherin, anti-β-catenin, anti-VEGFR1-C-terminus and anti-VEGFR2-C-terminus, and then subsequent Western blot analyzed using these four antibodies, respectively. (**a**) A panel of representative Western blots. (**b**) Band intensities of replicate experiments (n = 4 independent experiments) were quantified as described in the Methods and regression analysis undertaken to assess the association of these proteins.

Immunoprecipitation and Western blot demonstrated a strong, but dynamic, association between VE cadherin, β-catenin and full length VEGFRs 1 and 2 (Fig. 6). In the untreated cells, VEGFR1 demonstrated stronger association with VE-cadherin and β-catenin than VEGFR2. PEDF alone did not affect this association. However, a decreased VEGFR-1 association with VE-cadherin and β-catenin was observed after one-hour VEGF treatment while the association of VEGFR-2 with VE-cadherin/β-catenin remained unchanged, thus reducing the VEGFR-1∶VEGFR-2 ratio. By 6 hr, sustained presence of VEGF further reduced VEGFR-1 association with VE-cadherin/β-catenin but increased VEGFR-2 which led to a further reduction in the VEGFR-1∶VEGFR-2 ratio. By 24r, VEGF treatment not only caused a dissociation of the VE-cadherin/β-catenin complex, but also an almost complete loss of both VEGFR-1 and VEGFR-2 association with VE-cadherin or β-catenin. Taken together, these data suggest that the ratio of VEGFR1∶VEGFR2 may be important in the integrity of AJs. VEGF-induced VEGFR/VE-cadherin/β-catenin dissociation could be blocked by adding PEDF and this could be reversed by γ-secretase inhibition (Fig. 6).

### PEDF regulates VE-cadherin phosphorylation

**Figure 7:**
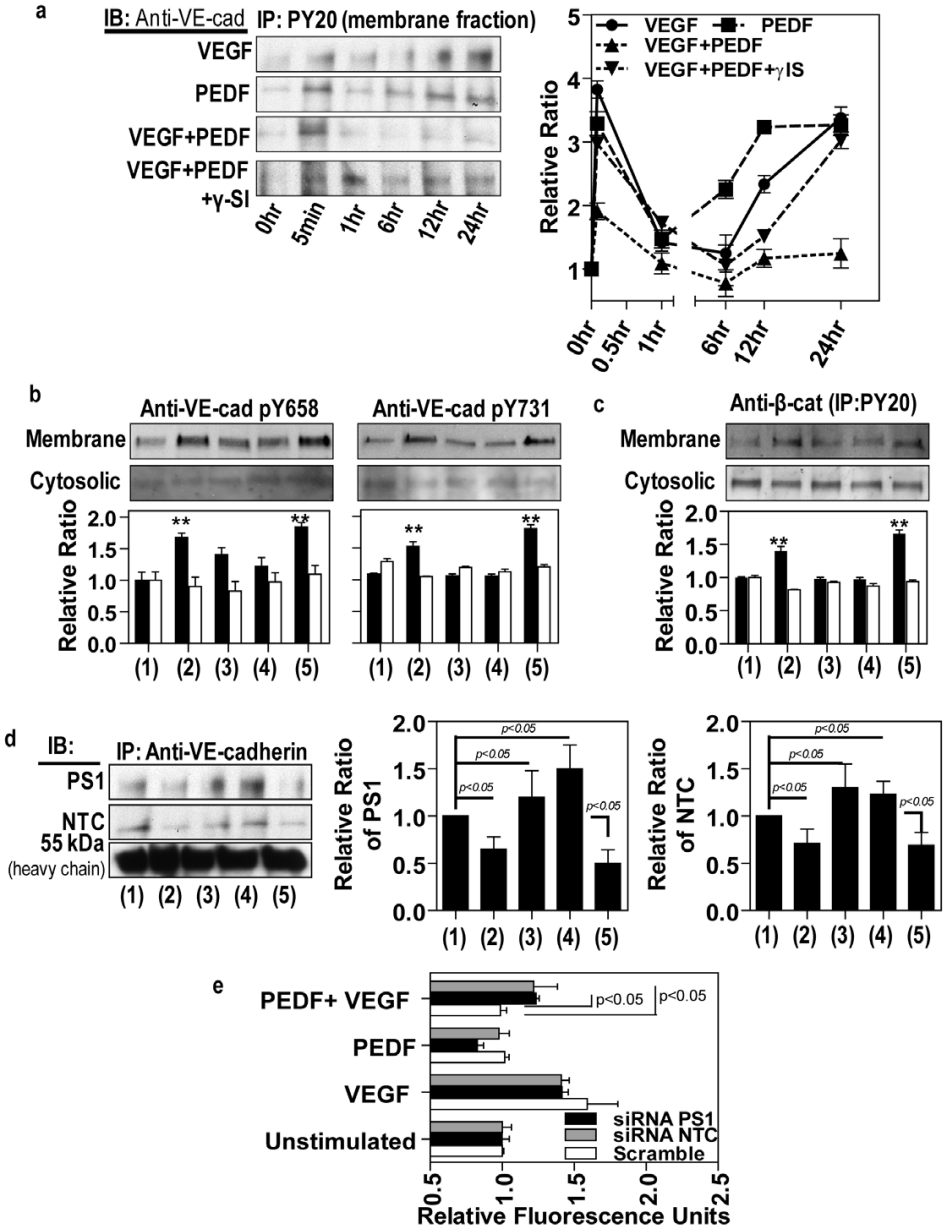
PEDF inhibits VEGF-induced phosphorylation of membrane-bound AJ proteins and promotes association of presenilin and nicastrin with VE-cadherin. Confluent retinal endothelial cells were either unstimulated (1) treated with VEGF alone (2), PEDF alone (3), VEGF followed by PEDF 6 hours later without (4) and with (5) γ-secretase inhibitor over 24 hours. VEGFA and PEDF were used at 100 ng/ml and γ-secretase inhibitor at 1 nM. Cells were separated into membrane and cytosolic fractions followed by immunoprecipitation. (**a**) Total VE-cadherin levels were determined by immunoprecipitation of the membrane fraction with anti-PY20 and Western blots for VE-cadherin. A panel of representative Western blots is shown on left and band intensities of replicate experiments (n = 4) on the right. (**b**) Western blot analysis to assess the effect of the different treatments on tyrosine phosphorylation of VE-cadherin at sites Y658 and Y731 (n = 4 independent experiments). (**c**) Total phosphorylation of β-catenin was assessed by immunoprecipitation of the membrane fraction with anti-PY20 and Western blots for β-catenin. (n = 4) (**d**) The association of presenilin-1 (PS1) and nicastrin (NTC) were confirmed by immunoprecipitation with anti-VE-cadherin, followed by Western blot analysis using anti-presenilin-1 and nicastrin. (**e**) To further confirm a role for γ-secretase in regulating vascular permeability presenilin-1 or nicastrin were knocked down with by transfection with AAV 2 expressing PS1 and NTC siRNAs. Scrambled siRNA acted as a control. Paracellular macromolecular permeability to 40 kDa Dextran-FITC was determined in cells treated with VEGF, PEDF, VEGF+PEDF and compared to unstimulated control. Densitometry analyses in (a), (c) and (d) are shown as the relative ratio of phosphorylated pVE-cadherin, pβ-catenin, presenilin-1 and nicastrin to heavy IgG chains. Densitometry in b is shown as the relative ratio to α-tubulin. The data are mean ± SEM. *p<0.05, **p<0.01 versus unstimulated group.

Since many studies have implicated tyrosine phosphorylation of VE-cadherin in modulation of endothelial permeability we investigated the effect of PEDF on VE-cadherin phosphorylation. As shown in Fig. 7a, VEGF-induced a rapid increase in tyrosine phosphorylation of VE-cadherin in the membrane fraction, which could be prevented by PEDF. A recent site directed mutagenesis study showed that mutation of pY658 or pY731 in VE-cadherin attenuated VEGF-induced permeability, suggesting that the regulation of endothelium permeability by VEGF requires the phosphorylation of VE-cadherin at tyrosine 658 and 731 [48]. As expected, VEGF induced a significant increase in the levels of pY658 and pY731 in the protein samples of the membrane factions (Fig. 7b). However, cytosolic VE-cadherin was not phosphorylated at pY658 and pY731 even though immunoprecitation with PY20 and subsequent Western blot for VE-cadherin showed strong tyrosine phosphorylation of VE-cadherin within cytosolic fractions (data not shown). Furthermore, PEDF inhibited the effects of VEGF and this was reversed by addition of a γ-secretase inhibitor (Fig. 7b). This was confirmed by Western blot using antibodies against VE-cadherin pY658 and pY731. The total tyrosine phosphorylation of membrane-associated β-catenin showed a very similar pattern to that of VE-cadherin upon treatment with VEGF, PEDF and γ-secretase inhibitor (Fig. 7c).

### Presenilin-1 and nicastrin associate with VE-cadherin and regulate vascular permeability

We confirmed a relationship between γ-secretase and VE-cadherin using both Western blot and siRNA knockdown (Fig. 7 d,e). The major γ-secretase components, presenilin-1 (PS1) and nicastrin (NCT), showed a strong association with VE-cadherin and this was significantly decreased following exposure of cells to VEGF (Fig. 7d). PEDF prevented VEGF-induced dissociation and interestingly PEDF was able to significantly increase these associations even without VEGF present. The effects of PEDF were prevented by a γ-secretase inhibitor. Furthermore, siRNA knockdown of PS1 and NTC was able to reduce the inhibitory effect of PEDF on VEGF-induced permeability of endothelial cells (Fig. 7e).

### Hypoxia increases vascular permeability

**Figure 8:**
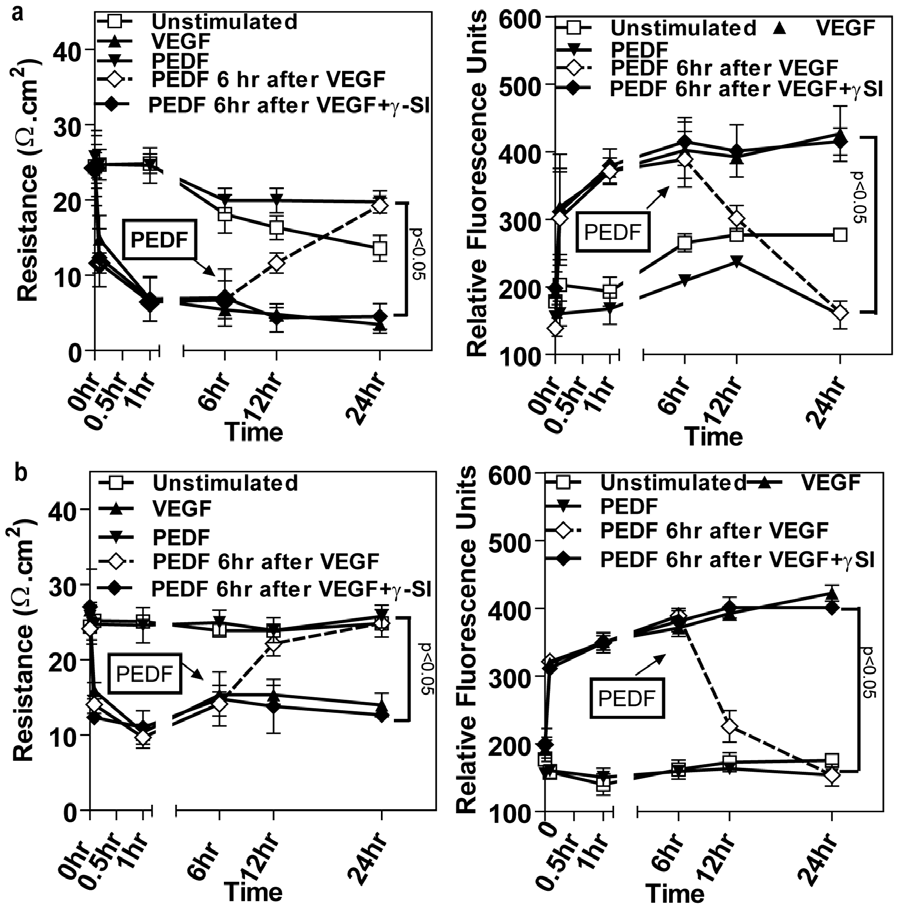
Hypoxia increases vascular permeability. Temporal changes in transendothelial resistance across a microvascular endothelial monolayer grown on a Transwell insert treated with vehicle (unstimulated), VEGFA alone, PEDF alone, simultaneous VEGFA+PEDF, PEDF 6 hours post VEGF and PEDF 6 hours post VEGF+γ-secretase inhibitor (γ-SI) under either (**a**) hypoxia or (**b**) retinal normoxia. (n = 4 independent experiments). VEGFA and PEDF were used at 100 ng/ml and γ-secretase inhibitor at 1 nM. In the case of PEDF 6 hours post VEGF, Transwell inserts were transferred to new wells containing basal medium without fluorescent dextran. Data are respresented as means± SEM.

Since angiogenesis normally occurs in a hypoxic environment we next examined to what extent junctional integrity was dependent on the local oxygen environment. We chose three oxygen environments based on our previous studies [49]; retinal hypoxia, inner retinal normoxia and standard incubator conditions. Under hypoxic conditions, vascular permeability demonstrated a gradual but significant (p<0.05) decrease in TER and increase in transendothelial flux in the absence of exogenous growth factors compared with cultures maintained under normoxia or standard incubator conditions (Fig. 8, Fig. 1a,b). VEGF-induced permeability was significantly increased under hypoxia compared to the other oxygen environments. Interestingly, PEDF had a greater inhibitory effect on VEGF-induced permeability under hypoxia than either normoxia or standard incubator conditions and in all cases this was completely inhibited by blocking γ-secretase (Fig. 8, Fig. 1).

### Hypoxia increases the expression of VEGF receptors and γ-secretase along with VEGFR dissociation from AJ proteins

**Figure 9:**
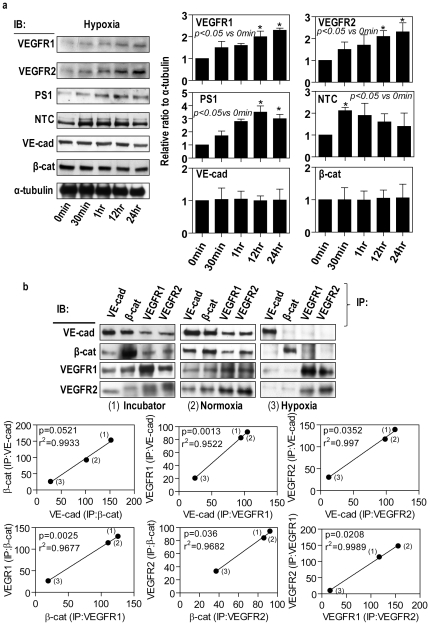
Hypoxia increases the expression of VEGF receptors and γ-secretase along with VEGFR dissociation from AJ proteins. (**a**) Confluent retinal microvascular endothelial cells were exposed to hypoxia for up to 24 hours. Cell lysated were examined by Western blot for VEGFR1, VEGFR2, presenilin-1 (PS-1), nicastrin (NCT), VE-cadherin and β-catenin. α-tubulin acted as the house keeping control. A representative Western blot is shown together with densitometric analysis from three separate experiments. (**b**) Confluent retinal microvascular endothelial cells were maintained under standard incubator conditions, retinal normoxia and hypoxia for 24 hours. Total cell lysates were split into four equal portions and immunoprecipitated with anti-VE-cadherin, anti-β-catenin, anti-VEGFR1-C-terminus and anti-VEGFR2-C-terminus, and then subsequent Western blot analyzed using these four antibodies, respectively. A panel of representative Western blots is shown. Band intensities of replicate experiments (n = 4) were quantified as described in the Methods and regression analysis undertaken to assess the association of these proteins.

It is well recognized that hypoxic conditions have profound effects on the endothelial junctions [50], [51]. Western blot demonstrated that hypoxia induced a time-dependent increase in VEGFR-1, VEGFR-2, presenilin-1 and nicastrin but had no significant effect on the expression of VE-cadherin and β-catenin (Fig. 9a) compared to normoxic and incubator conditions. To further characterize the effect of hypoxia we used immunoprecipitation and Western Blot to determine the association between VEGF receptors and AJ proteins. Hypoxia caused a significant decrease in the association of VE cadherin with β-catenin and the VE-cadherin complex with VEGFR-1 and VEGFR-2 compared to normoxia (Fig. 9b).

## Discussion

Tightly regulated vascular permeability plays a major role in vascular homeostasis. However, increased vascular permeability is a hallmark of numerous ocular conditions including diabetic retinopathy [4], [52], [53]. Increased leakiness of retinal vessels is apparent in both early, non-proliferative diabetic retinopathy as well as late stage, proliferative diabetic retinopathy with ensuing macular edema. Breakdown of the blood retinal barrier during diabetes has a strong association with increased VEGF levels and numerous VEGF-blocking strategies have been shown to reduce retinal vascular permeability [4]. PEDF, by contrast, functions as a potent anti-vasopermeability factor and acts primarily by blocking the biological actions of VEGF [39], [40]. However, since PEDF levels are reduced and VEGF levels increased in diabetic retinopathy [4], [44] this favors increased vascular permeability. Thus our observation that PEDF blocks VEGF-induced vascular permeability in vitro and in vivo is in keeping with the published literature [37], [38], [40], [41], [54]. However, PEDF maximally reduced VEGF-induced permeability if applied 6 hours following VEGF rather than PEDF given simultaneously with VEGF. Certainly this effect is not specific to PEDF as we have previously reported a more profound effect for placenta growth factor-1 (PlGF-1) on VEGF-induced permeability [55]. We believe that the cells need to be primed with VEGF before they can respond fully to PEDF inhibition and utilize VE-cadherin to stabilize the endothelial junctions. Furthermore, there is increasing evidence that growth factor levels, including VEGF [56], fluctuate within microenvironments and thus the order of exposure may be a critical factor that determines cellular response.

Tight junctions (TJs) and adherens junctions (AJs) play a major role in the control of vascular permeability and this barrier function requires the expression and organization of VE-cadherin and claudin-5, which are essential components of adherens junctions (AJs) and tight junctions (TJs) respectively [9], [10] in both the blood-brain and blood-retinal barriers. Cells require AJ formation to build TJs [57], [58] and recent reports indicate that co-ordinated disruption of VE-cadherin intracellular interactions culminates in the restructuring of both AJs and TJs and the subsequent opening of endothelial cell-cell junctions [9], [10], [59]. Our results demonstrate that VEGF leads to a rapid dissociation of AJs within about 15 minutes while changes in tight junctions are not observed until significantly later. PEDF acts to stabilize these junctional complexes, and in the case of exposure to PEDF following VEGF is able to rapidly reverse VEGF-induced dissociation of junctional complexes which occurs in AJ's prior to TJ's. This supports reports that co-ordinated disruption of VE-cadherin intracellular interactions culminates in the restructuring of both AJs and TJs and the subsequent opening of endothelial cell-cell junctions [9], [10], [59].

AJs are a complex of vascular endothelial-cadherin (VE-cadherin), α-catenin and β-catenin [1]. VE-cadherin is a transmembrane protein that plays a crucial role in the formation and maintenance of AJs. β- and γ-catenins can bind to cytoplasmic tail of VE-cadherin and link it to α-catenin, which in turn binds to the actin cytoskeleton [1], [8]. Tyrosine phosphorylation of VE-cadherin and other AJ proteins typically weaken cell-cell adhesions and increase vascular permeability [8]. VEGF-induced a rapid increase in tyrosine phosphorylation of VE-cadherin and that this occurred primarily at tyrosine 658 and 731 as previously reported [48]. PEDF blocked VEGF-induced total phosphorylation of membrane VE-cadherin as well as phosphorylation at Y658 and Y731. Tyrosine phosphorylation of membrane-associated β-catenin showed a very similar pattern to that of VE-cadherin upon treatment with VEGF and PEDF. Reports to date indicate that PEDF may prevent VEGF-induced permeability by inhibiting Src kinase activity [38], blocking VEGFR-2 activated phosphorylation of the MAP kinase/β-catenin signaling pathway [41] and blockade of urokinase plasminogen activator [41] which, via proteolytic degradation of VE-cadherin, alters the blood retinal barrier [60]. Our study identifies a novel, γ-secretase-dependent pathway, by which PEDF can regulate vascular permeability via translocation of VEGFRs. Both in vitro and in vivo inhibition of γ-secretase abrogated the beneficial effects of PEDF on VEGF-induced vascular permeability. This is in keeping with our previous observations that PEDF acts as a potent inhibitor of VEGF-induced angiogenesis by a γ-secretase-dependent translocation of both full length VEGFR and a cleaved intracellular domain of VEGFR-1 [24]. In this study we show a dynamic, association between AJ proteins and full length VEGFR-1 and VEGFR-2. Our data would indicate that the ratio of VEGFR1∶VEGFR2 may be important in maintaining the integrity of AJs. In untreated cells, VEGFR1 demonstrates stronger association with AJs than VEGFR2 while, in contrast, within 1 hour of VEGF treatment the ratio is reversed in favor of VEGFR2 association with AJs. Following 24 hour treatment with VEGF there is no association with AJ proteins, presumably because the AJs are fully dissociated by this time. PEDF prevented the change in VEGFR1∶VEGFR-2 ratio and the dissociation of AJs, moreover, this change in ratio was dependent on γ-secretase activity. VEGFR-2 is associated with VE-cadheren at AJs and VEGFR-2 may be responsible for the direct phosphorylation of AJ proteins [8], [61]. Furthermore, we have recently reported that neutralization of VEGFR-2 significantly decreases VEGF-induced phosphorylation of VE-cadherin while neutralization of VEGFR-1 induced phosphorylation of VE-cadherin [55]. This is in keeping with the current concept that VEGFR-1 is a potent negative regulator of VEGFR-2. However, it should be noted that not all retinal cells may behave the same since PEDF appears to prevent VEGF-mediated permeability in RPE cells by γ-secretase-dependent processing of VEGFR-2 [62].

Cells require AJ formation to build TJs [57], [58] and co-ordinated disruption of VE-cadherin intracellular interactions culminates in the restructuring of both AJs and TJs [9], [10], [59]. Furthermore, VE-cadherin regulates both claudin-5 expression and phosphorylation [9], [59], [63]. These observations are in keeping with our findings that changes in TJ integrity proceed AJ changes and that AJs re-associated before TJs. These key observations would indicate that AJs may offer a better therapeutic target when attempting to prevent or reverse increased retinal permeability and intraretinal leakage.

The association between both presenilin-1 and nicastrin with VE-cadherin is intriguing and could be responsible for either processing of VEGFRs or AJ proteins. With respect to AJ proteins, γ-secretase cleavage of both N-cadherin or E-cadherin can lead to disassembly of AJs and an intracellular signalling cascade [64], [65]. However, our studies would indicate that this may not be the case for endothelial cells since VEGF decreased γ-secretase association with VE-cadherin while PEDF prevented this and thus supports processing of VEGFRs as the critical positive regulator of AJ stabilization.

Although tissue hypoxia is regarded as a major factor in the pathogenesis of diabetic retinopathy there is little known about how this can affect the integrity of AJs. In the absence of exogenous growth factors vascular permeability gradually increased under hypoxic conditions, presumably due to the increase in angiogenic factors such as VEGF. Hypoxia caused a significant decrease in the association of VE cadherin with β-catenin, and VEGFRs further emphasizing that hypoxia facilitates increased vascular permeability. Furthermore, hypoxia in untreated cells induced a time-dependent upregulation of VEGFRs, presenilin-1 and nicastrin but not VE-cadherin and β-catenin compared to normoxia. Interestingly, exogenous PEDF had a greater inhibitory effect on VEGF-induced permeability under hypoxia than normoxia. This is unlikely to be due to increased PEDF levels since PEDF is downregulated under hypoxia.

In conclusion, we have shown that a) PEDF regulates VEGF-induced vascular permeability via a novel γ-secretase dependent pathway, b) the ratio of VEGFR-1∶VEGFR-2 is important in determining the degree of vascular permeability and c) hypoxia makes microvascular endothelial cells more susceptible to increased vascular permeability. Our studies would suggest that targeting downstream effectors of PEDF, such as VEGFR translocation and/or processing or γ-secretase activity may represent a promising therapeutic strategy for the treatment of diabetic vascular complications in the eye and other organs.

## Materials and Methods

### Materials

Recombinant VEGF165 was purchased from R&D systems (R&D Systems, Minneapolis, MN, USA) and PEDF from BioProducts MD (BioProducts MD, Middletown, MD, USA). γ-secretase inhibitors L685485 and DAPT were obtained from Sigma (Sigma, St. Louis, MO, USA). Anti-VE-cadherin antibody was obtained from Cell Signaling (Cell Signaling, Danvers, MA, USA), while anti-VE-cadherin pY568 and 731 antibodies were purchased from Invitrogen (Invitrogen, Carlsbad, CA). Both anti- β-catenin and α-tubulin antibodies were from Abcam (Abcam, Cambridge, MA, USA). Anti-presenilin 1 and nicastrin antibodies were obtained from Santa Cruz (Santa Cruz Biotechnology, Santa Cruz, CA, USA).

### Cell culture

Bovine retinal microvascular endothelial cells were isolated as previously described [24]. In brief, bovine retinas in ice cold Eagle's minimal essential medium (MEM) with HEPES were homogenized and microvessels trapped on an 83 µm nylon mesh. Vessels were transferred to a cocktail containing 500 µg/ml collagenase, 200 µg/ml pronase and 200 µg/ml DNase at 37°C for 20 min. The resultant vessel fragments were trapped on 53 µm mesh, washed with MEM and centrifuged at 225 g for 10 min. The pellet was resuspended in microvascular endothelial cell basal medium (MCDB131) with growth supplement (Invitrogen, CA) at 37°C, 5% CO_2_ for 3 days. Purity was confirmed by Factor VIII and VE cadherin staining. Cells were used between passage 1 and 3 and all experiments were undertaken under standard incubator conditions unless otherwise stated. For permeability studies, endothelial cells were maintained at confluence for 3–4 weeks to allow establishment of junctional complexes and a transendothelial resistance of >20 Ω.cm^2^.

### Growth factor treatment

Confluent endothelial cell cultures were rendered quiescent for 45 min in serum-free basal medium VEGF-A and PEDF (alone or in combination) were added at 100 ng/ml based on our previous studies [24] and in the sequences indicated in the text for different time intervals. We extrapolated from this to select our choice of doses for the in vivo studies. Experiments were undertaken in the presence or absence of 1 nM γ-secretase inhibitors L685485 or DAPT (Sigma). Cells were analyzed at varying time periods as described below.

### Regulation of the oxygen environment

Three experimental oxygen environments were achieved using oxygen controlled glove boxes attached via a central airlock with O_2_, CO_2_, N_2_, temperature and humidity control (Coy Laboratory Products Inc., Grass lake, MI): hypoxia, pO_2_ = 5 mmHg; normoxia for the inner retina, pO_2_ = 40 mmHg; standard incubator conditions, pO_2_ = 135 mmHg [49]. All media and solutions were pre-equilibrated for 24 hours at the appropriate oxygen concentration prior to the beginning of the experiment. Confluent retinal microvascular endothelial cells in 6-well plates or on Transwell inserts were exposed to growth factors as described above and processed at different time points for TER and Western Blot analysis as described below.

### *In vitro* permeability measurements

Endothelial cells were maintained at confluence on porous polyester membrane inserts (6.5 mm diameter, 0.4 µm pore size; Transwell, Corning, Cambridge, MA) for up to four weeks prior to experimentation. The upper and lower compartments contained 100 µl and 0.5 ml of media, respectively. Experimental treatments with growth factors and/or γ-secretase inhibitor as described above were added to the upper compartment.

#### Transendothelial Resistance (TER) Measurement

TER measurements were performed using an EVOM volt-ohmmeter (World Precision Instruments, Sarasota, FL). At the indicated time intervals, resistance readings (Ω) were obtained from each insert and multiplied by the membrane area (Ω×cm^2^) to obtain values of TER. The resistance value of an empty culture insert (no cells) was subtracted. Data were collected from triplicate inserts per treatment in each of three separate experiments.

#### Paracellular permeability assay

The growth medium in the upper chamber was replaced with 100 µl of growth medium containing a 1 mg/ml FITC-dextran 20 or 40 and the cells allowed to equilibrate at 37°C for 15 min. Twenty µl of stock growth factors and/or γ-secretase inhibitor were added to the medium to achieve the concentrations and combinations as described above. In the case of addition of PEDF 6 hours following VEGF, Transwell inserts were transferred to new wells containing basal medium without fluorescent dextran. A 50 µl sample of medium was taken in triplicate from the lower chamber at various times and placed in a 96-well cluster plates to measure fluorescent intensity (excitation at 530 nm and emission at 590 nm). In all cases the volume of the basal chamber was maintained at 500 µl by replacement of the 50 µl sample with 50 µl of fresh medium.

### *In vivo* retinal permeability assay

All animal studies were performed under a protocol approved by the Institutional Animal Care and Use Committee at the University of Florida, (IACUC Study #201005197), and in accordance with the ARVO Statement for the Use of Animals in Ophthalmic and Vision Research. In vivo retinal vascular permeability was assessed as previously described [55]. Eight-week-old C57BL/6 mice were purchased from Jackson Laboratories (Bar Harbor, ME). Mice received the following intravitreal injections (1 µl) with a 32-gauge needle into one eye: VEGF; PEDF; VEGF plus PEDF together; 0.9% saline vehicle; VEGF followed by PEDF 6 hours later; VEGF plus γ-secretase inhibitor (L685485 or DAPT); PEDF plus γ-secretase inhibitor; VEGF plus PEDF plus γ-secretase inhibitor; VEGF followed by 0.9% saline 6 hours later. Based on previous titration studies (data not shown) test compounds were given at the following concentration, VEGF 80 ng/µl; PEDF (80 ng/µl); L685485 24 ng/µl; DAPT 16 ng/µl. 46 hours post the first injection mice received tail vein injections of FITC-labeled albumin (0.5 mg in 50 µl vehicle). The mice were placed on heating pads to maintain normal body temperature. After 2 hours the mice were treated in two ways: 1) For albumin leakage measurements animals were killed and the eye that received the intravitreal injection enucleated and the retinas removed and placed in PBS. The retina were rinsed in buffer, disrupted mechanically, cleared by centrifugation and the FITC-albumin in the supernatant fraction was quantified against a standard curve of FITC-albumin using a spectrofluorometer as previously described [55]. 2) For histology and immunostaining the mice were perfused with 4% paraformaldehyde by cardiac puncture prior to enucleation of the eyes.

### Immunocytochemistry

#### Cell cultures

Bovine retinal microvascular endothelial cells were cultured on glass bottom microwell dishes (MatTek Corporation, MA) coated with attachment factor. At confluence cells were fixed with 4% paraformaldehyde plus 4% sucrose in PBS and permeabilized with 0.1% Triton X-100. Non-specific staining was blocked with 5% BSA in PBS for 30 min at room temperature. The cells were then dual labelled with goat polyclonal anti-VE-cadherin antibody (Santa Cruz) and rabbit polyclonal anti-Claudin-5 ( Abcam, MA) all at 1∶1000 in PBS containing 5% BSA at 4°C overnight. The secondary antibodies Alexa Fluor 549-labeled donkey anti-goat IgG (Invitrogen, CA) for VE-cadherin and Alexa Fluor 488-labeled donkey anti-rabbit IgG (Invitrogen, CA) for Claudin-5, all at 1∶1000 in 5% BSA in PBS at room temperature for 1 hour in the dark. Exclusion of the primary antibody acted as the negative control. Staining was evaluated and digital photomicrographs obtained using an Olympus IX81-DSU Spinning Disk confocal microscope.

#### Retinal flat mounts

Retina from the mouse studies were carefully removed from the eyecup, permeabilized with 0.2% Triton X-100 and non-specific binding blocked by 10% normal goat serum in PBS overnight at 4°C. The retinas were then transferred to a solution of primary antibody and incubated for 24 hours at 4°C. The primary antibodies were rabbit anti-VE-Cadherin (1∶100, Cell Signaling Technology, Inc., Danvers, MA, USA) and rabbit anti-Claudin-5 (1∶3000, Abcam Inc., Cambridge, MA, USA). The retinas were transferred to the secondary antibody for 24 hours at 4°C after washing in PBS with 0.2% Triton X-100. The secondary antibody was Cy3 conjugated goat anti-rabbit IgG (1∶250). To specifically label the vascular endothelium, the retinas were then incubated 30 minutes at room temperature in 1∶500 FITC-conjugated agglutinin in 10 mM HEPES, 150 mM NaCl and 0.1% Tween 20. Exclusion of the primary antibody acted as the negative control. Retinas were flat mounted onto microscope slides and covered in aqueous VectaShield mounting medium (Vector Laboratories, Inc., Burlingame, CA) for observation by confocal microscopy. Digital confocal images were captured with an Olympus DSU-Olympus IX81 confocal microscope (Olympus America, Inc., Center Valley, PA, USA). Digital images from experimental and control retinas with identical photomultiplier tube gain settings were captured by using imaging software-SlideBook ^4.2.^. Maximum projections generated from z-section stacks of confocal images were processed identically in experimental and control retinas.

### Immunoprecipitation and Western blotting

Different subcellular fractions (membrane and cytosolic) were prepared from microvascular endothelial cells receiving the different treatments using a Subcellular Protein Extraction Kit according to the manufacturer's instructions (Calbiochem, San Diego, CA). Purity of fractions was confirmed by Western Blot using antibodies against pan-cadherin (membrane); histone 1 (nuclear); pan-cytokeratin (cytoskeleton); calpain (cytosolic).

For immunoprecipitation, individual capture antibody (2 µg/ml) was mixed with 250 µL of cell lysates for 1–2 h at 4°C. Protein A/G plus Agarose was added (20 µl) and agitated overnight at 4°C. Agarose beads were washed 3 times and resuspended in PBS. The samples were boiled for 5 min and immunocomplexes were resolved on SDS-polyacrylamide gels (7.5%–12%) and subjected to Western blotting.

For Western blotting, 30 µg of cell lysate was electrophoresed on 7.5%–12% SDS-polyacrylamide gels under reducing conditions for VE-cadherin, β-catenin, VEGFR-1, and VEGFR-2, presenilin-1, nicastrin, PY20 (polyclonal antibodies, Santa Cruz Biotechnology), Claudin-5, VE-cadherin (pY658 and pY731) (polyclonal antibodies, Abcam Inc.) After electrophoresis, proteins were transferred onto nitrocellulose membranes (Bio-Rad) and nonspecific binding sites were blocked with 10% non-fat dry milk in PBS. Blots were probed with the above antibodies used at a concentration of 0.1 µg/ml, followed by an HRP-conjugated secondary antibody. The membranes were incubated with ECL Plus Western blotting detection system (Amersham Biosciences, Piscataway, NJ) and exposed to Biomax MR film (Sigma). Band intensities were determined using NIH Image.

### Protein association studies

To determine if there is association of β-catenin, VE-cadherin, VEGFR-1 and VEGFR-2 during VEGF/PEDF/VEGF+PEDF-induced signaling, the cells were treated with growth factors as described above. Then 1 ml of cell lysate containing 500 µg/ml protein for each treatment was divided into four equal portions. The four portions were immunoprecipitated with either rabbit anti- β-catenin, VE-cadherin, VEGFR-1 and VEGFR-2 antibodies. For control, preimmune sera were used to replace the primary antibodies. After Western blotting analysis with the same four antibodies, the band intensity was determined by NIH Image and regression analysis undertaken as previously described [47].

### Statistics

All experiments were repeated at least three times. The TER and paracellular permeability data at different time points were assessed using a Student's *t* test plus ANOVA for multiple comparisons. The Mann-Whitney test was used to determine statistical significance in the data of protein expression obtained using Western blotting analysis. Results are expressed as mean±standard error of the mean. Statistical analysis was performed using Prism 5 (GraphPad Software, La Jolla, CA) with *p*<0.05 considered statistically significant.
